# Investigation into Critical Gut Microbes Influencing Intramuscular Fat Deposition in Min Pigs

**DOI:** 10.3390/ani14213123

**Published:** 2024-10-30

**Authors:** Long Jin, Ke Li, Zhimin Li, Xuankai Huang, Li Wang, Xibiao Wang, Shengwei Di, Shiquan Cui, Yuan Xu

**Affiliations:** College of Animal Science and Technology, Northeast Agricultural University, No. 600 Changjiang Road, Xiangfang District, Harbin 150030, China; a18963123464@163.com (L.J.); abc540409376@126.com (K.L.); m18647960742@163.com (Z.L.); hxk87822@163.com (X.H.); wanglisy1982@126.com (L.W.); wangxibiao@neau.edu.cn (X.W.); dishengwei@neau.edu.cn (S.D.)

**Keywords:** Min pig, 16S rRNA sequencing, gut microbes, IMF

## Abstract

To determine the pivotal microorganisms affecting intramuscular fat (IMF) accumulation in Min pigs and to discern the extent of the influence exerted by various intestinal segments on IMF-related traits, we sequenced 16S rRNA from the contents of six intestinal segments from a high IMF group (Group H) and a low IMF group (Group L) of Min pigs weighing 90 ± 1 kg. We then compared their diversity and disparities in bacterial genera. Group H exhibited considerably higher α diversity in the jejunum and colon than Group L (*p* < 0.05). When 95% confidence levels were considered, the main β diversity components for the ileum, caecum, and colon within Groups H and L exhibited absolute segregation. Accordingly, 31 differentially abundant genera across Group H were pinpointed via LEfSe and the Wilcoxon test (*p* < 0.05) and subsequently scrutinised based on their distribution and abundance across distinct intestinal segments and their correlation with IMF phenotypes. The abundances of *Terrisporobacter*, *Acetitomaculum*, *Bacteroides*, *Fibrobacter*, *Treponema*, *Akkermansia*, *Blautia*, *Clostridium sensu stricto 1*, *Turicibacter*, *Subdoligranulum*, the *[Eubacterium] siraeum group*, and *dgA 11 gut groups* were positively correlated with IMF content (*p* < 0.05), whereas those of *Bacillus*, the *Lachnospiraceae NK4A136 group*, *Streptococcus*, *Roseburia*, *Solobacterium*, *Veillonella*, *Lactobacillus*, the *Rikenellaceae RC9 gut group*, *Anaerovibrio*, and the *Lachnospiraceae AC2044 group* were negatively associated with IMF content (*p* < 0.05). Employing PICRUSt2 for predicting intergenic metabolic pathways that differ among intestinal microbial communities revealed that within the 95% confidence interval the colonic microbiome was enriched with the most metabolic pathways, including those related to lipid metabolism. The diversity results, bacterial genus distributions, and metabolic pathway disparities revealed the colonic segment as an influential region for IMF deposition.

## Simple Summary

The meat quality of pork is significantly associated with its intramuscular fat (IMF) content. In recent years, the associations of gut microbes with fat deposition in various animals have been well-documented. However, studies on intestinal microbes typically utilise faecal samples as a proxy. Given that the composition and diversity of microorganisms vary significantly across different gastrointestinal segments, faecal samples may not accurately reflect the spatial distribution of microbial species among distinct intestinal regions. This research relied on microbial 16S rRNA sequencing to identify influential intestinal microflora categories impacting the IMF content in six distinct intestinal segments (duodenum, jejunum, ileum, caecum, colon, and rectum) and pinpointed the primary segment influencing the IMF content. These findings provide a theoretical foundation for modulating gut microbes to increase pork quality and the IMF content.

## 1. Introduction

The Min pig is a pig breed with a rich historical background that is indigenous to the frigid conditions of China’s northeastern and northern regions. It is renowned for its exceptional cold resistance, disease resistance, and superior meat quality. Across the spectrum of factors impacting pork quality, the intramuscular fat (IMF) content is paramount. The greater the IMF content, the greater the pork’s water-holding capacity and marbling score [1]. The IMF content of Min pigs is greater than that of commercial pig breeds, representing a significant factor contributing to the exceptional flavour of Min pork compared with commercial pork. Thus, a thorough comprehension of the factors influencing the IMF content of Min pigs is crucial for optimising pork quality and increasing economic benefits.

IMF primarily resides in the perimysial and endomysial membranes, and glycerol triesters and phospholipids are significant components. IMF and skeletal muscle both originate from mesodermal somatic mesenchymal stem cells (MSCs) [2]. During the differentiation of MSCs, most cells differentiate into myogenic progenitor cells (MPS), which undergo a series of proliferation and differentiation processes to form myotubes, which then fuse into muscle fibres to form muscle [3,4]. A smaller proportion of cells differentiate into fibro-adipogenic progenitor cells (FAPs), which further differentiate into preadipocytes. Under the regulation of transcription factors, cell cycle factors, and other proteins, these preadipocytes proliferate and differentiate into mature adipocytes through intermediate stages, ultimately forming IMF [5,6]. Numerous factors are instrumental in influencing IMF content, including genetic predisposition, sex, and dietary energy levels. For example, feeding pigs a diet deficient in lysine or supplementing the diet with conjugated linoleic acid (CLA) can significantly increase the IMF content of the longissimus dorsi muscle [7,8,9,10]. Several investigations have identified pivotal candidate genes influencing IMF content, such as *PPARs*, *C/EBPs*, *FAS*, *LPL*, *ATGL*, and *FABPs*, among others [11,12,13,14,15]. Additionally, in a study of Tibetan chickens, *SFRP5*, *FGF1*, and *FGF10* were found to be positively correlated with IMF deposition [16]. The *JAZF1* gene has been shown to be associated with lipid metabolism in sheep [17]. SNPs of the *DGAT1*, *FABP4*, *LEP*, *RORC*, and *SCD1* genes have a significant impact on intramuscular fat deposition in cattle [18].

Additionally, studies have revealed the critical role of gut microbes in lipid deposition. The immense population of gut microbes, reaching 10^14^ entities [19], is composed primarily of anaerobic and facultative anaerobic bacteria, which are intricately linked with host metabolism, growth, immunity, disease, and health, earning the gut microbiome the title of “the overlooked organ” [20,21]. The most prevalent phyla among the gut microbes include Firmicutes, Bacteroidetes, Proteobacteria, Actinobacteria, Verrucomicrobia, and Fusobacteria. Among these taxa, the phyla Bacteroidetes and Firmicutes are the most prevalent [22], and fluctuations in their relative abundance potentially influence fat deposition levels. In studies of mouse models, compared with germ-free mice, bacteria-colonised mice (CONV-Rs) exhibited increased fat deposition levels [23]. Research has revealed a substantial regulatory role of gut microbes in various aspects, such as skeletal muscle development, subcutaneous fat and IMF deposition, and skeletal muscle metabolism, in pigs [24,25,26]. In poultry, through whole-genome sequencing and 16S rRNA sequencing, it has been established that gut microbes play crucial roles in chicken abdominal fat deposition and metabolic regulation, potentially via host genetics [27,28].

Researchers are currently investigating the colonisation dynamics and altering factors of porcine intestinal microorganisms via nutritional modulation and faecal microbiota transplantation strategies [29,30,31]. However, most experimental studies utilise faecal swabs over other biological samples for analysis. Hence, few studies have investigated the differences in bacterial community composition and abundance among different sections (duodenum, jejunum, ileum, caecum, colon, and rectum) of the pig gut and the extent to which each section influences phenotypic traits. We identified the Min pig as the subject of our examination in this study. The experimental groups were established on the basis of the IMF content level in the pigs (high IMF group (Group H) and low IMF group (Group L)). Through comparisons of bacterial genera and abundance profiles across six intestinal segments between Groups H and L, we intended to investigate the latent effect of the microbial diversity of each intestinal segment on IMF deposition in Min pigs. The aim of this study is to elucidate the internal regulatory mechanisms and pathways of this phenomenon.

## 2. Materials and Methods

### 2.1. Animal Feeding and Husbandry

The experimental piglets were raised at a pig farm in Lanxi County, Heilongjiang Province. Before rearing, the pig house was thoroughly cleaned and disinfected. A total of 92 piglets (43 castrated boars and 49 sows) from the same batch of neonatal pigs were selected for rearing in the same pen. The average birth weight of the piglets was 1.06 ± 0.17 kg. All of the piglets were weaned at 35 days of age, with an average weaning weight of 9.14 ± 1.43 kg. Before weaning, the piglets were housed in farrowing pens with the sow. After weaning, they were transferred to the nursery barn for group rearing. Once the piglets reached a weight of 25 ± 2 kg, they were moved to the finishing barn. After weaning, the pigs were fed twice daily with unrestricted access to water, and the nutritional data for the diet are as follows (Table 1). The temperature and humidity of the pig house were monitored daily to maintain appropriate conditions. The pig house and experimental piglets were regularly disinfected, and water and feed cleanliness were maintained. No antibiotics or probiotics were used during the trial period. The pigs were raised until 220–240 days of age, reaching an average body weight of 91.15 ± 5.96 kg. Within the pig population, pigs whose weight reached 90 ± 1 kg and whose age and health conditions were similar were selected for further analysis. A total of 30 pigs (including 14 castrated boars and 16 sows) were selected. Food and water intake were withheld 24 h prior to slaughter.

### 2.2. Sample Collection

Samples of the contents of the duodenum (D), jejunum (J), ileum (I), caecum (Ce), colon (Co), and rectum (R) of each pig were collected within 45 min post slaughter, placed in 2 mL cryovials, and immediately transferred to a liquid nitrogen tank for transport and preservation in a −80 °C freezer. The sampling process was conducted on ice. The sample collection points for intestinal contents were taken from the midsection of each intestinal segment to avoid mixing and crossover of contents from adjacent regions. Samples of longissimus dorsi muscles were collected, placed in 2 mL cryovials, immersed in a liquid nitrogen tank for transport, and preserved in a −80 °C freezer for use in preparing frozen sections for oil red O staining. Three samples of longissimus dorsi muscle weighing approximately 20 g (m_0_) from each pig were also collected and preserved at −20 °C for determination of the IMF content. All procedures were performed in accordance with the experimental animal care and usage guidelines established by the Chinese Ministry of Agriculture.

### 2.3. Measurement of the IMF Content

The IMF content was determined via the acidic hydrolysis method. Each longissimus dorsi sample was encapsulated in a freeze-drier (SCIENTZ-12N, Ningbo Scientz Biotechnology Co., Ltd., Ningbo, China) for 72 h and subsequently crushed into powder. The powder was inserted into a filter bag, which was then sealed and weighed (m_1_). The sealed filter bag was placed in an acid degradation apparatus (ANKOM HCI hydrolysis system, ANKOM Technology Co., Ltd., Macedon, NY, USA), exposed to 500 mL 3 mol/L hydrochloric acid, heated to 90 °C for 60 min, and rinsed for 20 min to liberate the fat bound to or sequestered within the tissue. After acid hydrolysis, the sample was dried for 2 h; then, a fat extraction apparatus (ANKOM XT15, ANKOM Technology Co., Ltd., Macedon, NY, USA) was utilised to extract the fat with petroleum ether at 90 °C for 80 min, ensuring that the fat in the sample was fully dissolved in petroleum ether. The filter bag was placed in an oven to dry until a constant weight was reached, after which it was weighed (m_2_). The calculation methodology for the IMF content is as follows:

IMF content = (m_1_ − m_2_)/m_0_ × 100%

Relying on the IMF content measurement results, the high IMF group (Group H) and the low IMF group (Group L) were separated. A cryostat slicer was used to cut back longissimus muscle samples from the H and the L Groups. The sections were stained with oil red O, and morphological observations were conducted to assess the distribution of IMF.

### 2.4. Microbial DNA Extraction and 16S rRNA Sequencing

In accordance with the IMF content data clustering analysis, six pigs from each group (three castrated boars and three sows) were ultimately selected for 16S rRNA sequencing of their duodenal (D), jejunal (J), ileal (I), caecal (Ce), colon (Co), and rectal (R) content samples, for a total of 72 samples. Pig intestinal contents’ DNA was extracted via a T Guide S96 magnetic bead method with a faecal genomic DNA extraction kit (TianGen Biochemical Technology (Beijing) Co., Ltd., Beijing, China, DP812). The concentration of the extracted DNA was determined via an enzyme analyser (Gene Compang Limited, synergy HTX, Hong Kong, China), followed by amplification. The integrity of the PCR product was examined via 1.8% agarose gel electrophoresis, and purification was performed using VAHTSTM DNA Clean Beads (Vazyme Biotech Co., Ltd., Nanjing, China) magnetic beads. Quantification was performed via ImageJ 1.4.3.67 software (NIH, Bethesda, Rockville, MD, USA).

Primers were designed according to a fixed region within the sequence of microbial ribosomal RNA or a specific gene fragment of the microorganism. The universal primer was appended with universal sequencing adapters and sample-specific tag sequences. PCR amplification was performed on the V3 + V4 variable regions of the 16S rRNA gene via primers 338F (5′-ACTCCTACGGGAGGCAGCA-3′) and 806R (5′- GGACTACHVGGGTWTCTAAT-3′). The product was purified, quantified, and homogenised to form a sequencing library. The library that passed quality inspection was sequenced using an Illumina NovaSeq 6000 (Illumina, Inc., San Diego, CA, USA). The raw image data file obtained from sequencing was transformed into original sequences (sequenced reads) through base calling analysis and stored in FASTQ format.

### 2.5. Statistical Analysis

The raw reads obtained from sequencing were subjected to filtering via Trimmomatic V0.33 software; subsequently, the primer sequence was identified and removed via Cutadapt 1.9.1 software to yield clean reads devoid of the primer sequence. The dada2 [32] algorithm within QIIME 2020.6 [33] was utilised for noise reduction, double-end sequence alignment, and removal of chimeric sequences to generate operational taxonomic units (OTUs). Random sequences were sampled to create a rarefaction curve [34] to assess if the sequencing data adequately reflect species diversity. The Shannon diversity index was plotted using Mothur 1.34.4 and R 3.1.1 tools to show microbial diversity at different sequencing depths. Venn diagram analysis [35] using the R tools was used to illustrate the number of shared and unique features among the samples. The SILVA 138 [36] reference database was employed to classify the feature sequences via a naïve Bayes classifier, which provides community composition statistics at the phylum and genus levels. R language tools were utilised to construct sample community structure diagrams. The α and β diversity indices of the samples were evaluated via QIIME 2020.6 software. The species richness was assessed using the Chao1 and Ace indices, while species diversity was evaluated using the Shannon and Simpson indices. Principal Coordinate Analysis (PCoA) using weighted UniFrac, an algorithm in QIIME, was employed to calculate the distance between samples. The Python 2.7.8 linear discriminant analysis (LDA) effect size (LEfSe) 1.1.1 package [37] was used to analyse the distribution map of the LDA values of the differentially abundant microorganisms. The Wilcoxon test methodology was used to identify the differentially abundant bacterial genera across different groups. PICRUSt2 v2.3.0 was used to predict the functional gene expression differences in metabolic pathways among the microbial communities of different groups of samples.

## 3. Results

### 3.1. Phenotype Grouping and Morphological Analysis

**Figure 1:**
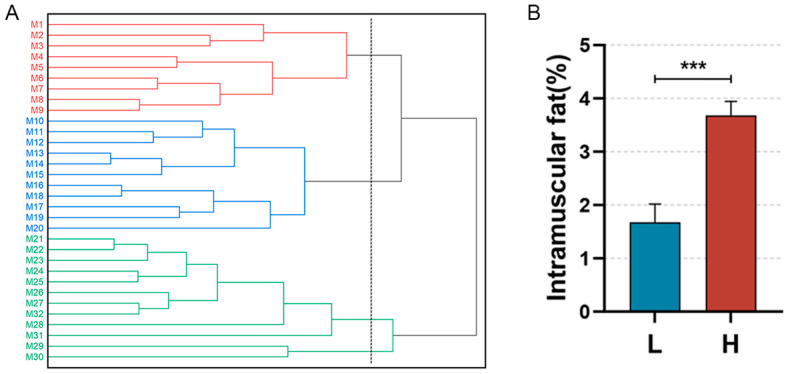
Sample clustering and comparison of IMF content. (**A**): Conceptual clustering diagram for the Min pig population on the basis of IMF content. Red lines (M1–M9): individuals in the low IMF group; Blue lines (M10–M20): individuals with intermediate IMF levels; Green lines (M21–M30): individuals in the high IMF group. (**B**): Difference in IMF contents between Groups L and H, *** *p*< 0.0001.

**Figure 2:**
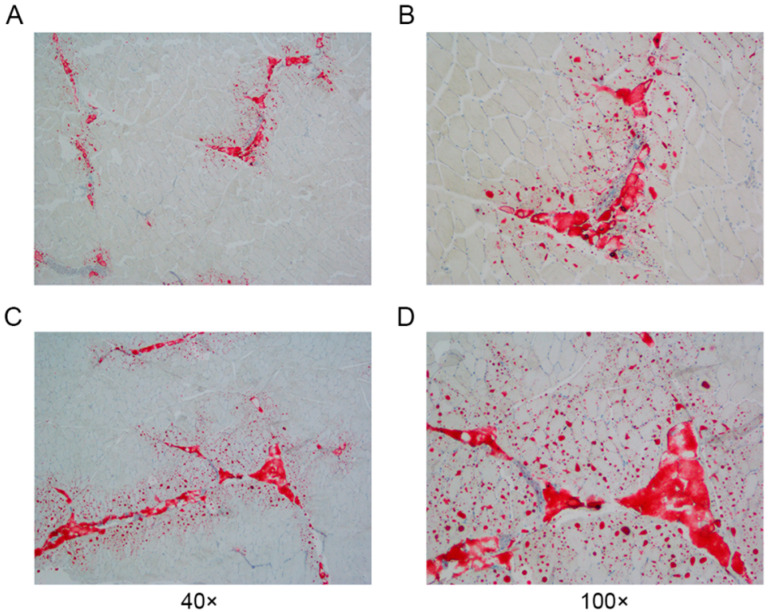
Paraspinal longissimus muscle oil-red-O-stained sections from Groups L and H. (**A**,**B**): Section from Group L. (**C**,**D**): Section from Group H.

A systematic cluster analysis was conducted on the 30 slaughtered pigs (M1–M30) on the basis of their body weights and IMF contents. The clustering results are depicted below (Figure 1B). The population is clustered into three groups (M1–M9, M10–M20, and M21–M30), with M1–M9 classified as the low IMF group (Group L) and M21-M30 as the high IMF group (Group H). The difference in the IMF content between the two groups was highly significant (*p* < 0.001) (Figure 1B). We observed the distribution of IMF in the sample sections via oil red O staining (Figure 2).

### 3.2. 16S rRNA Sequencing and α, β Diversity Analysis

The sequenced samples were divided into a total of 12 groups on the basis of the IMF content level and distinct intestinal segments. HD: Group H duodenal content samples; LD: Group L duodenal content samples; HJ: Group H jejunal content samples; LJ: Group L jejunal content samples; HI: Group H ileal content samples; LI: Group L ileal content samples; HCe: Group H caecal content samples; LCe: Group L caecal content samples; HCo: Group H colon content samples; LCo: Group L colon content samples; HR: Group H rectal content samples; and LR: Group L rectal content samples.

**Figure 3:**
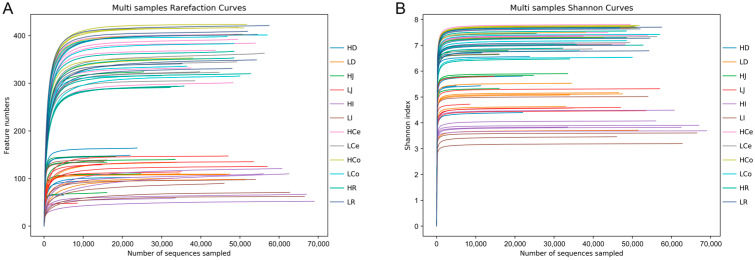
Sequencing depth chart of the entire sample. (**A**): Dilution curve. (**B**): Shannon curve.

**Figure 4:**
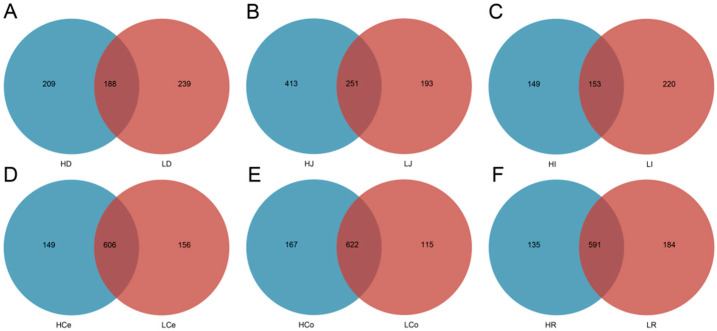
The exclusive and shared OTU taxa among the contents of different intestinal segments in Groups H and L. (**A**): Duodenum. (**B**): Jejunum. (**C**): Ileum. (**D**): Caecum. (**E**): Colon. (**F**): Rectum.

The sequencing operation resulted in a total of 5,288,516 paired reads. After quality control and assembly, 4,861,722 clean reads were generated, and each sample yielded at least 39,745 clean reads for an average of 67,524 reads per sample. The dada2 method was employed to eliminate noise from the sequence data, and the sequencing depth was assessed through dilution curves and Shannon index curves (Figure 3). The curve results indicated that all of the samples in each group entered a plateau phase. This signifies that at the current sequencing depth, we can detect the vast majority of species in the samples, satisfying subsequent analytical requirements. A total of 1582 OTUs were obtained from all of the samples for an average of 237 per sample. The number of shared and unique OTUs among different intestinal segments in each group is illustrated in a Venn diagram (Figure 4).

**Figure 5:**
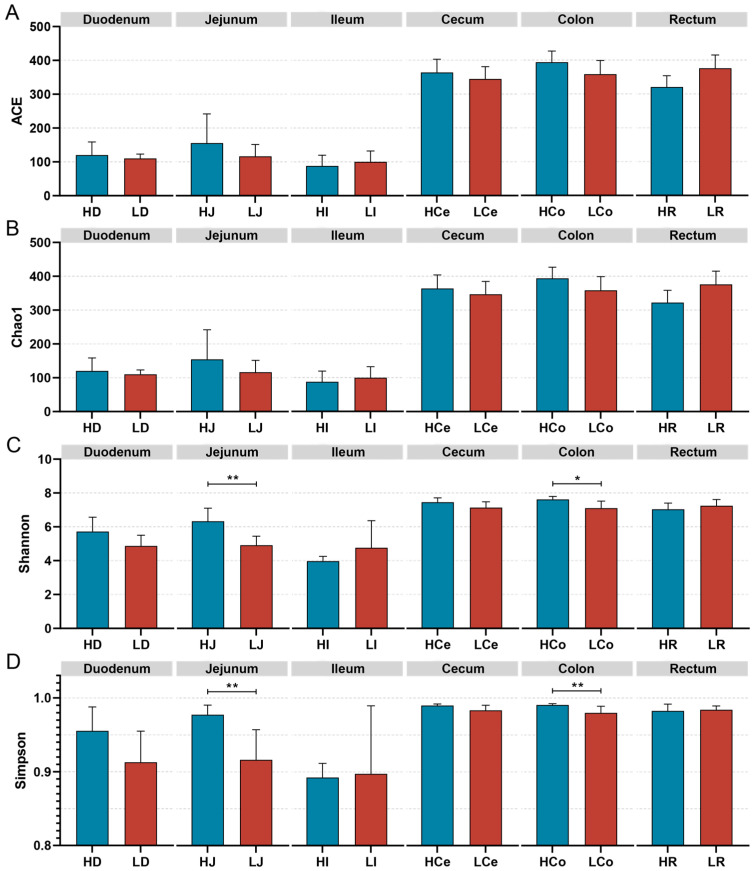
The α diversity indices of specific intestinal segments from Groups H and L. (**A**): ACE index. (**B**): Chao1 index. (**C**): Shannon index. (**D**): Simpson index, * *p* < 0.05, ** *p* < 0.01.

The α diversity of the samples was measured via four indices: the Chao1, Ace, Shannon, and Simpson indices (Figure 5). Species richness was assessed via the Ace and Chao1 indices, whereas species diversity was evaluated via the Shannon and Simpson indices. The findings indicated that the differences in the ACE index and the Chao1 index between Group H and Group L in each intestinal segment were not significant; however, the Simpson index and the Shannon index for the jejunum and colon samples significantly differed between the H and L Groups. Specifically, the Simpson and Shannon indices in the jejunum were significantly greater in Group H than in Group L (*p* < 0.01); in the colon, the Group H Simpson index was significantly greater than that of Group L (*p* < 0.01), and the Shannon index was significantly greater than that of Group L (*p* < 0.05). No significant differences were observed in the Shannon or Simpson indices of the other intestinal segments.

**Figure 6:**
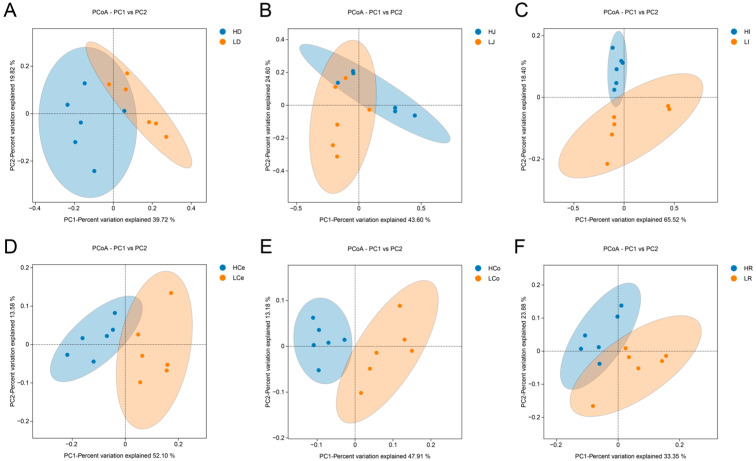
β diversity indices of the respective intestinal segments in Groups H and L. (**A**): Duodenum. (**B**): Jejunum. (**C**): Ileum. (**D**): Caecum. (**E**): Colon. (**F**): Rectum.

β diversity among groups of samples is depicted via a PCoA plot (Figure 6). The first principal component (PC1) accounted for 39.72% of the variance in the duodenal samples, whereas the second principal component (PC2) contributed 19.82%. In the jejunum, the first and second principal components contributed 43.60% and 24.60%, respectively. The first and second principal components of the ileum accounted for 65.52% and 18.40%, respectively. The first and second principal components of the caecum contributed 52.10% and 13.58%, respectively. The first and second principal components of the colon contributed 47.91% and 13.18%, respectively. The first and second principal components of the rectum contributed 33.35% and 23.88%, respectively. The Group H and Group L duodenal, jejunal, and rectal samples did not demonstrate complete separation of the principal components, indicating a certain degree of similarity between these segments of the two groups. The principal components of the ileum, caecum, and colon samples in Group H and Group L were completely separated within the 95% confidence interval, indicating that there was a significant difference in β diversity between Group H and Group L in these three intestinal segments.

### 3.3. Microbial Composition and Differential Analysis of Various Intestinal Segments

**Figure 7:**
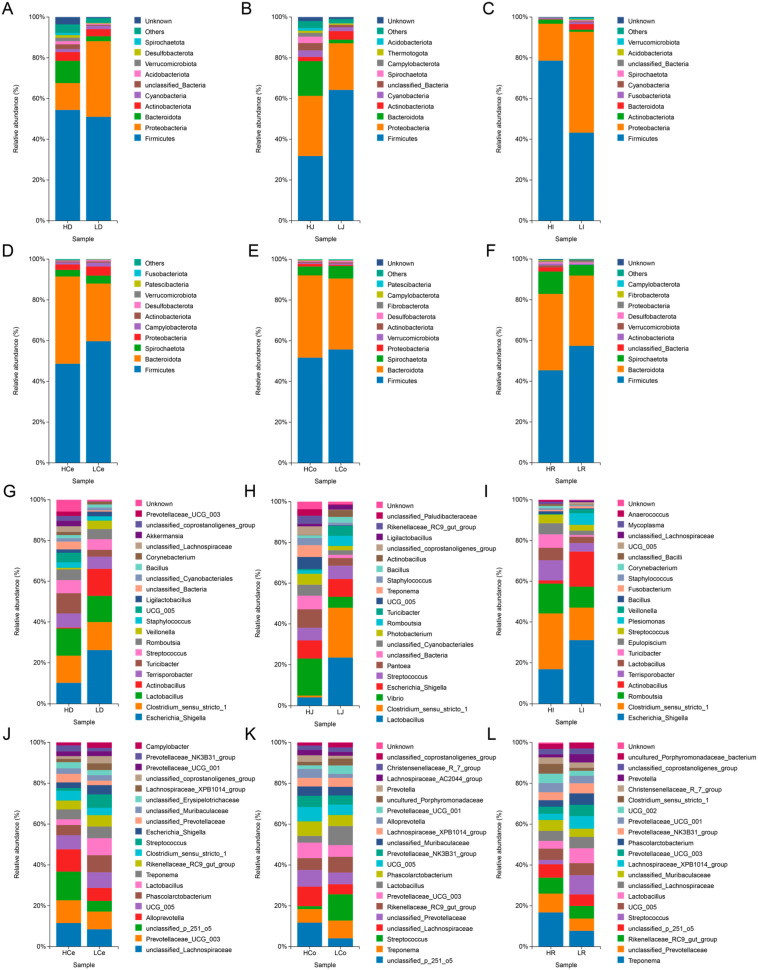
Distribution of prominent microbial populations at both the phylum and genus levels for each intestinal segment. (**A**–**F**): Phylum level. (**G**–**L**): Genus level.

To ascertain the composition and disparities of the intestinal microorganisms in Groups H and L at the phylum and genus levels, we examined the abundance of these intestinal microorganisms (Figure 7).

At the phylum level, the dominant taxa in the small intestine segment exhibit substantial spatial disparity within different intestinal segments, whereas those in the large intestine segment exhibit superior consistency. The abundance of Firmicutes and Proteobacteria was highest in the small intestine segment, and the relative abundance of these two phyla accounted for more than 60% and 90%, respectively, in the ileum. Compared with that in Group L, the abundance of Firmicutes in Group H was lower in the jejunum but greater in the duodenum and ileum; the abundance of Proteobacteria was greater in the jejunum but lower in the duodenum and ileum; and the abundance of Bacteroidota was greater in the duodenum and jejunum but lower in the ileum. In the large intestine segment, the abundances of Firmicutes and Bacteroidota were the highest, with the relative abundances of these two phyla accounting for more than 80% of the total bacteria. Compared with those in Group L, the abundances of Firmicutes in Group H were lower, the abundances of Bacteroidota were greater, and the ratios of the abundances of Firmicutes and Bacteroidota were lower than those in Group L.

At the genus level, *Escherichia* and *Shigella* were the predominant genera in the duodenum and ileum segments of the small intestine, whereas Lactobacillus was dominant in the jejunum. These observations revealed that the abundances of these genera in Group H were lower than those of Group L. *Clostridium sensu stricto 1* was a dominant genus in the duodenum, jejunum, and ileum, and other genera exhibited varying abundances across different groups. Across the large intestinal segments, *Lachnospiraceae*, *p 251 o5*, and *Treponema* were the most abundant genera in the caecum, colon, and rectum, respectively, with Group H abundances consistently higher than those in Group L. The distribution of genera within each segment of the small intestine also exhibited spatial variation, with only 7 out of the top 20 most abundant genera appearing in all three intestinal segments. The distribution of genera within each segment of the large intestine was consistent, and 12 out of the top 20 most abundant genera appeared in all three intestinal segments.

**Figure 8:**
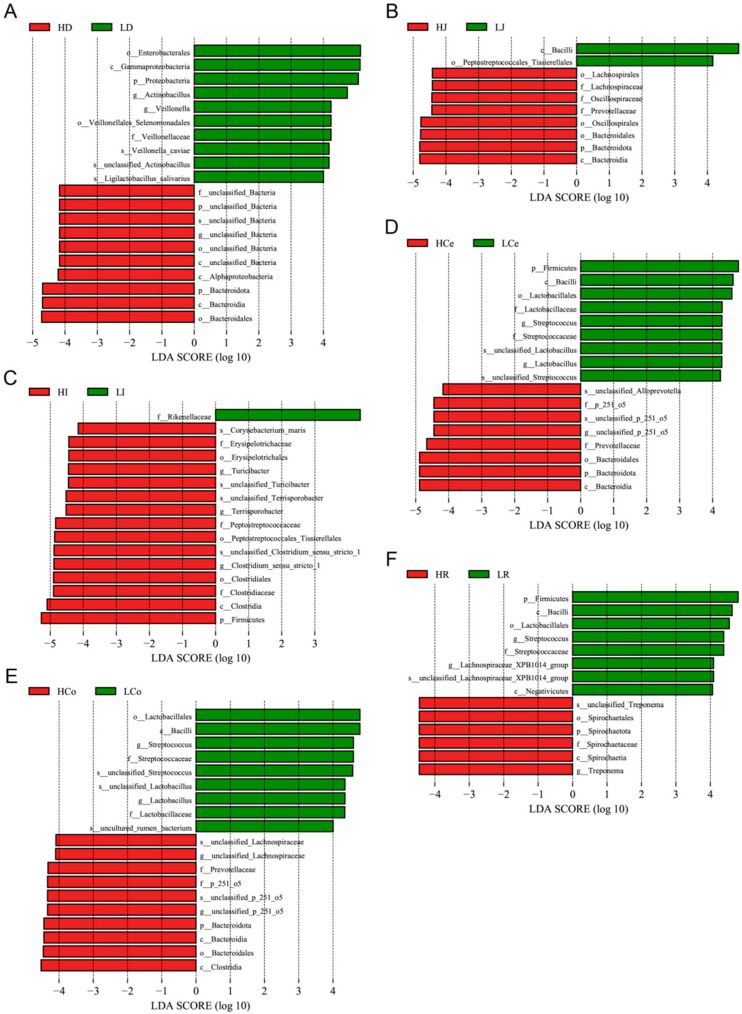
The microbiome profile analysis results for each intestinal segment (using an LDA > 4.0 as a discernment criterion). (**A**): Duodenum. (**B**): Jejunum. (**C**): Ileum. (**D**): Caecum. (**E**): Colon. (**F**): Rectum.

We performed LEfSe analysis on our samples, gauging the extent of LDA effects and comparing relative microbial abundance disparities at various taxonomic levels from phylum to species (Figure 8). Upon eliminating redundant calculations of identical microorganisms across different taxonomic levels, we identified four dominant duodenal bacteria in Group H and nine in Group L; six dominant jejunal bacteria in Group H and two in Group L; ten dominant ileal bacteria in Group H and one in Group L; four dominant caecal bacteria in Group H and seven in Group L; six dominant colon bacteria in Group H and six in Group L; and two dominant rectal bacteria in Group H and six in Group L.

**Figure 9:**
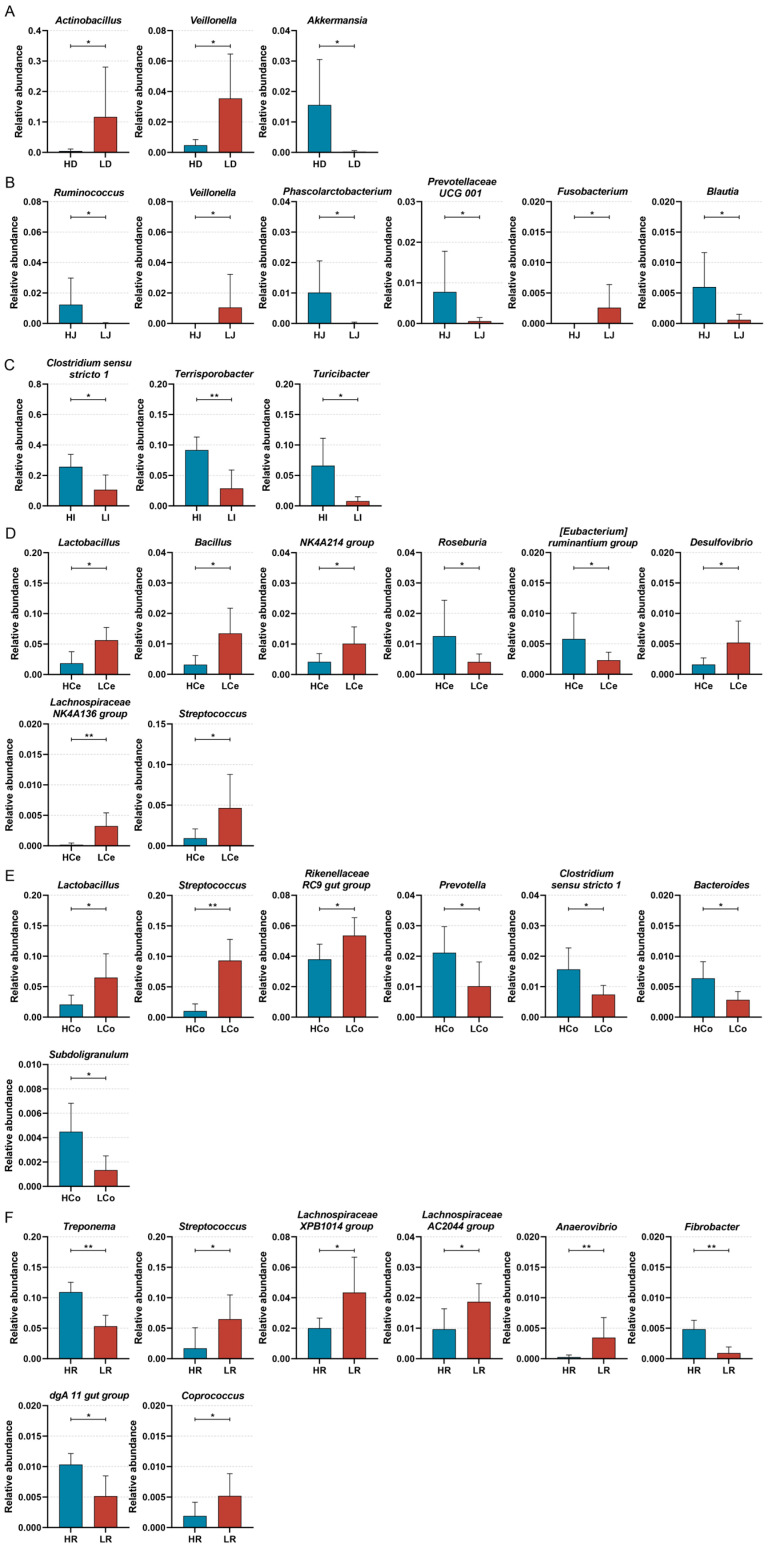
Significant differences were identified in the Group H and Group L genera across intestinal segments, * *p* < 0.05, ** *p* < 0.01. (**A**): Duodenum. (**B**): Jejunum. (**C**): Ileum. (**D**): Caecum. (**E**): Colon. (**F**): Rectum.

To discern the bacterial taxa that exert a notable influence on IMF content in Min pigs, we next subjected samples from each cohort to a Wilcoxon test analysis at the genus level (Figure 9). The results indicated that the abundances of *Actinobacillus* and *Veillonella* in the duodenum were markedly greater in Group L than in Group H, whereas *Akkermansia* presented a greater abundance in Group H (*p* < 0.05); in the jejunum, the abundances of *Veillonella* and *Fusobacterium* in Group L were significantly greater than those in Group H, and *Ruminococcus*, *Phascolarctobacterium*, *Prevotellaceae* UCG 001, and *Blautia* demonstrated a greater abundance in Group H (*p* < 0.05); in the ileum, each of the three distinctive bacterial genera was predominant in Group H; *Clostridium sensu stricto 1* and *Turicibacter* demonstrated a significant disparity in abundance (*p* < 0.05), and *Terrisporobacter* demonstrated an extraordinarily significant disparity in abundance (*p* < 0.01). In the caecum, *Lactobacillus*, *Bacillus*, the NK4A214 group, *Desulfovibrio*, the *Lachnospiraceae* NK4A136 group, and *Streptococcus* demonstrated a significantly greater abundance in Group L than in Group H. *Roseburia* and the *[Eubacterium] ruminantium* group demonstrated a significantly greater abundance in Group H than in Group L (*p* < 0.05); in the colon, *Lactobacillus*, the *Rikenellaceae* RC9 gut group, and *Prevotellaceae* UCG 001 demonstrated a significantly higher abundance in Group L than in Group H (*p* < 0.05). *Streptococcus* demonstrated an extraordinarily higher abundance in Group L than in Group H (*p* < 0.01). Prevotella, *Clostridium sensu stricto 1*, *Bacteroides*, and *Subdoligranulum* demonstrated significantly higher abundances in Group H than in Group L (*p* < 0.05); in the rectum, the abundance of Group L *Streptococcus*, the *Lachnospiraceae* XPB1014 group, *Prevotellaceae* UCG 003, the *Lachnospiraceae* AC2044 group, and *Coprococcus* was significantly higher than in Group H (*p* < 0.05). *Anaerovibrio* demonstrated an extraordinarily higher abundance in Group L than in Group H (*p* < 0.01). The abundance of the Group H *dgA* 11 gut group was significantly higher than in Group L (*p* < 0.05); *Treponema* and *Fibrobacter* demonstrated an extraordinarily higher abundance in Group H than in Group L (*p* < 0.01). The distinctive bacterial genera varied across each intestinal segment, showcasing the spatial disparity of bacterial genera in each intestinal segment.

To verify the correlation between taxonomic diversity and IMF content, we conducted a Pearson correlation analysis (Table 2). The correlations between differential bacterial genera and the IMF content exhibited considerable variability between the intestinal segments. *Terrisporobacter*, *Acetitomaculum*, *Bacteroides*, *Fibrobacter*, and *Treponema* abundances were extremely significantly positively correlated with the IMF content (*p* < 0.01); *Akkermansia*, *Blautia*, *Clostridium sensu stricto 1*, *Turicibacter*, *Subdoligranulum*, *[Eubacterium] siraeum group*, and *dgA* 11 gut group abundances were markedly positively correlated with the IMF content (*p* < 0.05); *Bacillus*, *Lachnospiraceae NK4A136 group*, *Streptococcus*, *Roseburia*, and *Solobacterium* abundances were extremely significantly negatively correlated with the IMF content (*p* < 0.01); and Veillonella, Lactobacillus, Rikenellaceae *RC9 gut group*, *Anaerovibrio*, and *Lachnospiraceae AC2044 group* abundances were notably negatively correlated with the IMF content (*p* < 0.05). The correlations of abundances of other bacterial genera with the IMF content were relatively weak.

### 3.4. Predictive Analysis of Community Function Genes

**Figure 10:**
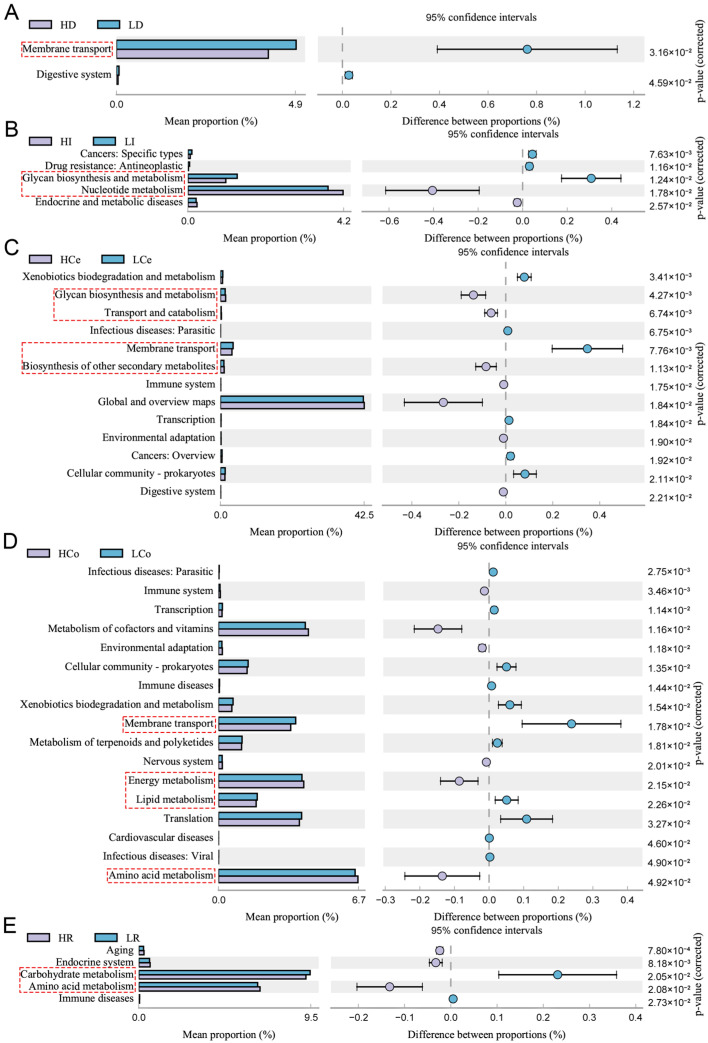
Genomic variations in the microbiome communities of Groups H and L revealed significant KEGG pathway enrichment (95% confidence interval), *p* < 0.05, Red dashed boxes indicate KEGG pathways related to metabolism. The circles show deviations from the 95% confidence interval, indicating inter-group differences. (**A**): duodenum, (**B**): ileum, (**C**): caecum, (**D**): colon, (**E**): rectum.

The 16S rRNA gene sequence of the sample was aligned with reference sequences from the Integrated Microbial Genomes (IMG) microbial genomic database via PICRUSt2. This aids in predicting KEGG pathway profiles of microbial communities among groups within samples (Figure 10). The intestinal segments failed to show statistical significance for group differences in the jejunum (*p* > 0.05); the duodenum was enriched predominantly in membrane transport pathways; the ileum was enriched predominantly in carbohydrate biogenesis and metabolism and nucleotide metabolism pathways; the caecum was enriched primarily in carbohydrate biogenesis and metabolism, transport and catabolic processes of glycolipids, membrane transport, and biosynthesis of other secondary metabolite pathways; the colon was significantly enriched in membrane transport, energy metabolism, lipid metabolism, and amino acid metabolism pathways; and the rectum was enriched predominantly in carbon metabolism and amino acid metabolism pathways. The bacterial enrichment pathways in each intestinal segment were associated with numerous metabolic pathways. The caecum and colon were enriched in the most metabolic-related pathways, and the colon was the only intestinal segment that exhibited significant differences in the activity of lipid metabolism pathways. The colonic microbes may play a more significant role in the synthesis and metabolism of IMF than those of other segments.

## 4. Discussion

The gut microbiome plays an essential role in porcine IMF deposition, and faecal microbe transplantation across various pig breeds has demonstrated that abundances of specific gut microorganisms can markedly increase the IMF levels and lipid metabolism status of the pig population [38], primarily through the metabolic products of different types of microorganisms, which can regulate nutrient absorption and degradation in pigs. For example, acetate can decrease cholesterol (TC) and triglyceride (TG) contents, and propionate can stimulate the expression of *SIRT1* mRNA in the longest muscle of the back (*p* < 0.05) [39,40]. In addition, alterations in the abundance of gut microorganisms can be induced by nutritional regulation and subsequently influence the levels of SCFAs in the intestine, thus increasing the IMF content [41].

The findings of this study demonstrate a marked divergence in the microbial assemblage distribution between the small intestinal and large intestinal segments. The similarity and redundancy of intestinal contents are not as prevalent in the large intestine as they are in the small intestine. In terms of bacterial community α diversity, the Simpson and Shannon indices of Group H and L species in the jejunum and the colon exhibited significant or extremely significant differences, indicating substantial species diversity discrepancies between Group H and Group L in the jejunum and the colon; the β diversity results revealed that the primary component differences between samples from the ileum, caecum, and colon in Group H and Group L were the greatest, and they were therefore capable of resulting in complete separation of the groups. By combining the α and β diversity results, it can be discerned that the microbial diversity of the colon exhibits considerable intergroup discrepancies, potentially harbouring a greater number of microorganisms that influence IMF deposition.

At the genus level, the results of the intergroup Wilcoxon test and correlation analysis revealed significant or extraordinarily significant correlations between the abundances of several genera and the IMF content. The roles of some genera in fat deposition or catabolic processes have been substantiated in recent studies. For example, *Bacteroides, Treponema*, and *Fibrobacter* abundance demonstrated robust positive correlations with IMF content, replicating the findings of Fang et al. [42]. *Akkermansia* presented a high abundance in rabbits with high IMF content, and its role in the production of acetic acid played a minor role in the synthesis process of IMF [43,44]. *Acetitomaculum* can similarly generate acetic acid from simple sugars [45]; *Blautia* abundance and that of its metabolites also appear to be positively correlated with IMF [46]. In this study, *Clostridium sensu stricto 1* was predominant in both the ileum and the colon of the H Group, which corroborated the conclusions of Tang et al. [47]. Feeding pigs low-protein diets could increase the IMF content of the longissimus dorsi muscle, with an increase in the relative abundances of *Terrisporobacter* and *Turicibacter* [48]. *Subdoligranulum* abundance plays a pivotal role in the microbial network of Peking ducks, demonstrating a robust positive correlation with the abundance of *Akkermansia*, and it is proficient in generating butyrate salt [49], which can be absorbed in the intestine and transported to muscle tissue through the bloodstream, activate the expression of IMF-related candidate genes, and promote the biosynthesis of IMF [50].

Among the bacterial genera whose abundances were negatively correlated with the IMF content, numerous species of the genera *Actinobacillus*, *Lactobacillus*, and *Bacillus* were detected in the caecum and colon. Experiments in mice demonstrated that bacilli can decrease fat deposition levels, resulting in anti-obesity effects [51,52]. The addition of *Bifidobacterium* to pig diets can increase the proportion of *Firmicutes* in the caecum and increase lipid metabolism levels [53]. *Streptococcus* exhibited significant abundance differences in both the colon and rectum, and its abundance in the colon was found to be extremely significantly negatively correlated with the IMF content. Research has indicated that *Streptococcus* can alter the composition of bile acids to reduce fat deposition levels [54]. *Anaerovibrio* abundance was negatively correlated with the IMF content in this study, and research by Bergamaschi M indicated that its abundance was negatively correlated with pig feed efficiency and backfat thickness [55]. RLS can decrease fat deposition levels in mice and increase *Roseburia* abundance [56], which is consistent with the findings of this study. A study on the rearing method of Tibetan black sheep demonstrated that compared with pasture grazing, indoor rearing tends to increase fat deposition levels, enhance meat quality, and decrease the abundance of the *Rikenellaceae RC9* gut group [57], indicating its negative correlation with fat deposition.

The findings from PICRUSt2 functional prediction indicate that diverse bacterial genera are involved in multiple metabolic processes and that the products of these metabolic processes can reach muscle through the microbial–intestinal–muscle axis, where they participate in biological synthesis and metabolism. According to the PICRUSt2 results, the caecum and colon sections were enriched with the most metabolic-related differentially expressed pathways, with each containing four pathways, while the average ratio of metabolic pathways enriched in the colon was the highest, indicating the highest contribution of colonic microorganisms to metabolism. The colon was the only intestinal section enriched in lipid metabolic-related pathways.

The results of this study indicate that the abundance of gut microbiota is closely associated with various metabolic pathways and plays a crucial role in the synthesis of IMF. Specifically, the beneficial genera in the colonic microbiota are more abundant and show a stronger correlation with lipid metabolism pathways. Considering the differences in microbial composition and diversity, we propose that the colon is a critical intestinal segment affecting IMF content. Future research could explore dietary interventions or microbiome modulation to enhance IMF deposition in pigs. Additionally, further screening and cultivation of key probiotics within the colonic microbiota could be pursued, as this would have significant implications for production efficiency and meat quality in the pork industry.

However, this study has several limitations. First, the sample size is relatively small, which may limit the generalisability of the findings. Additionally, the influence of environmental factors and feeding conditions on the microbiome has not been thoroughly examined. Future research should encompass larger sample sizes and comparative analyses of various management practices to improve the reliability of the results.

## 5. Conclusions

In this study, we subjected the intestinal contents of Min pigs with variable IMF contents to 16S rRNA sequencing and identified a total of 31 significantly differentially abundant bacterial genera. Through correlation analysis, we selected 22 bacterial genera that impacted the IMF characteristics of Min pigs: 12 were positively correlated with the IMF content, and 10 were negatively correlated. Concurrently, on the basis of the diversity, abundance of bacterial genera, and functional gene pathway enrichment results, we identified the colon as the principal intestinal segment influencing the IMF content of Min pigs, providing an advancement in the potential capacity to optimise IMF content by altering the composition of the colonic microbes.

## Figures and Tables

**Table 1:** Base ration formulation and nutrient levels.

	Crude Protein	Net Energy (MJ/kg)	Lysine	Total Calcium	Phytate Phosphate
Conservation period	17.5	10.68	1.4	0.6	0.35
Fattening period	15.5	10.06	1	0.5	0.25

**Table 2:** Pearson’s correlation coefficients (r) between the abundance of genera and the IMF content in each intestinal segment, * *p* < 0.05, ** *p* < 0.01.

Intestine Segment	Genus	r	Intestine Segment	Genus	r
Duodenum	Akkermansia	0.612 *	Colon	Acetitomaculum	0.717 **
	Veillonella	−0.594 *		Bacteroides	0.719 **
Jejunum	Blautia	0.586 *		Rikenellaceae RC9 gut group	−0.659 *
Ileum	Clostridium sensu stricto 1	0.613 *		Streptococcus	−0.746 **
	Terrisporobacter	0.765 **		Subdoligranulum	0.673 *
	Turicibacter	0.626 *		[Eubacterium] siraeum group	0.647 *
Caecum	Bacillus	−0.778 **	Rectum	Anaerovibrio	−0.608 *
	Lachnospiraceae NK4A136 group	−0.769 **		Fibrobacter	0.818 **
	Lactobacillus	−0.686 *		Lachnospiraceae AC2044 group	−0.67 *
				Roseburia	−0.774 **
				Solobacterium	−0.772 **
				Treponema	0.802 **
				dgA 11 gut group	0.686 *

## Data Availability

The data presented in this study are available upon request from the corresponding author. The availability of the data is restricted to investigators based in academic institutions.
